# Dose and Fractionation in Radiation Therapy of Curative Intent for Non-Small Cell Lung Cancer: Meta-Analysis of Randomized Trials

**DOI:** 10.1016/j.ijrobp.2016.07.022

**Published:** 2016-11-15

**Authors:** Johanna Ramroth, David J. Cutter, Sarah C. Darby, Geoff S. Higgins, Paul McGale, Mike Partridge, Carolyn W. Taylor

**Affiliations:** ∗ Nuffield Department of Population Health, University of Oxford, Oxford, Oxfordshire, UK; † Department of Oncology, University of Oxford, Oxford, Oxfordshire, UK; ‡ CRUK/MRC Oxford Institute for Radiation Oncology, Oxford, Oxfordshire, UK

## Abstract

**Purpose:**

The optimum dose and fractionation in radiation therapy of curative intent for non-small cell lung cancer remains uncertain. We undertook a published data meta-analysis of randomized trials to examine whether radiation therapy regimens with higher time-corrected biologically equivalent doses resulted in longer survival, either when given alone or when given with chemotherapy.

**Methods and Materials:**

Eligible studies were randomized comparisons of 2 or more radiation therapy regimens, with other treatments identical. Median survival ratios were calculated for each comparison and pooled.

**Results:**

3795 patients in 25 randomized comparisons of radiation therapy dose were studied. The median survival ratio, higher versus lower corrected dose, was 1.13 (95% confidence interval [CI] 1.04-1.22) when radiation therapy was given alone and 0.83 (95% CI 0.71-0.97) when it was given with concurrent chemotherapy (*P* for difference=.001). In comparisons of radiation therapy given alone, the survival benefit increased with increasing dose difference between randomized treatment arms (*P* for trend=.004). The benefit increased with increasing dose in the lower-dose arm (*P* for trend=.01) without reaching a level beyond which no further survival benefit was achieved. The survival benefit did not differ significantly between randomized comparisons where the higher-dose arm was hyperfractionated and those where it was not. There was heterogeneity in the median survival ratio by geographic region (*P*<.001), average age at randomization (*P*<.001), and year trial started (*P* for trend=.004), but not for proportion of patients with squamous cell carcinoma (*P*=.2).

**Conclusions:**

In trials with concurrent chemotherapy, higher radiation therapy doses resulted in poorer survival, possibly caused, at least in part, by high levels of toxicity. Where radiation therapy was given without chemotherapy, progressively higher radiation therapy doses resulted in progressively longer survival, and no upper dose level was found above which there was no further benefit. These findings support the consideration of further radiation therapy dose escalation trials, making use of modern treatment methods to reduce toxicity.

**Summary**

We conducted a meta-analysis of overall survival in 3795 patients with non-small cell lung cancer who were randomized in 21 trials comparing higher versus lower radiation therapy doses of curative intent. In trials with chemotherapy, higher radiation therapy doses led to poorer survival, but in trials where chemotherapy was not given, higher time-corrected biologically equivalent doses resulted in longer survival. These findings support consideration of further trials of radiation therapy dose escalation within the context of toxicity reduction.

## Introduction

Lung cancer is the most common cause of cancer death worldwide, and survival has improved little since the mid-1970s, with 10-year survival at only 4% in 2011 1, 2. Almost 90% of lung cancers are non-small cell (NSCLC) (3), and surgery is the main curative treatment. Many patients are, however, inoperable at presentation (4), and for them, radiation therapy of curative intent may be considered, possibly in conjunction with chemotherapy. Since the 1970s, conventional radiation therapy in NSCLC has been defined as 60 to 63 Gy in 1.8- to 2.0-Gy fractions 5, 6, 7, but several trials have considered alternatives. These include split-course radiation therapy (with a several-day break), hyperfractionation (multiple smaller daily doses), hypofractionation (fewer, larger-dose fractions), acceleration (delivering the same dose over a shorter period), or changing total dose while keeping the same dose per fraction. Changes to these parameters all affect the biologically effective dose (BED) and may alter tumor control probability.

No randomized trials of radiation therapy dose and fractionation in NSCLC have included more than 600 patients, and many include fewer than 200. Consequently, most have not, individually, had sufficient power to detect modest effects on survival that would be important clinically. Where significant effects have been seen in individual trials, they may reflect extremes in the play of chance. More powerful inferences can be obtained when the data from all trials addressing a particular question are combined in a meta-analysis. Several meta-analyses of trials comparing different radiation therapy regimens in NSCLC have been conducted 8, 9, 10, but none has evaluated the effect of radiation therapy in terms of different levels of BED, and none has included all the relevant trials.

Several randomized trials have addressed the role of chemotherapy in addition to radiation therapy in NSCLC 11, 12. A Cochrane review of 3752 patients in 25 trials concluded that concurrent chemoradiation therapy resulted in better survival than radiation therapy alone (hazard ratio [HR], chemotherapy vs not: HR 0.71, 95% CI 0.64-0.80) or radiation therapy with sequential chemotherapy (HR 0.74, 95% CI 0.62-0.89) (13). Therefore, over the past decade, this has become the standard of care for patients with locally advanced NSCLC. However, patients treated with chemoradiation therapy experience more toxicity than do those treated with radiation therapy alone, particularly when chemotherapy is given concurrently 7, 13. Thus, within the context of chemoradiation, radiation dose escalation may result in a level of toxicity that outweighs any benefit from improved tumor control.

Given the ongoing uncertainty regarding the optimal dose for curative-intent radiation therapy for NSCLC, and the high levels of toxicity, it is not surprising that radiation therapy practice varies 12, 14, 15. Recent advances are, however, enabling radiation therapy to be delivered with lower toxicity than previously, so knowledge regarding the optimal dose for curative-intent radiation therapy is becoming more important. We have, therefore, conducted a meta-analysis of published data from randomized trials comparing different radiation therapy regimens. Our aim was to examine whether radiation therapy regimens delivering a higher corrected dose increased the overall survival in curative-intent radiation therapy for NSCLC, either when given alone or with chemotherapy.

## Methods and Materials

### Literature search and selection criteria

The Embase database was searched, using variations of the terms radiation therapy dose fractionation, hyperfractionation, hypofractionation, accelerated radiation therapy, and lung cancer (Fig. E1; available online at www.redjournal.org). Studies starting between 1980 and April 28, 2015, were eligible if they included a randomized comparison of 2 or more external beam radiation therapy dose fractionation regimens, with other treatments identical in any arms compared. A trial using hospital numbers to randomize was excluded (16).

### Calculation of time-corrected equivalent dose in 2-Gy fractions

To compare different dose fractionation regimens, total doses were converted to time-corrected equivalent doses in 2-Gy fractions (EQD2T) as follows (17):

$$\text{EQD}2\text{T}=\frac{\text{BED}}{1+\frac{2}{\alpha /\beta }}$$

where *α/β* was assumed to be 10 and BED was defined as

$$\text{BED}=Nd\left[1+\frac{d}{\alpha /\beta }\right]-k\left(T-T_{delay}\right)$$

where *N*=number of fractions; *d* = dose per fraction; *k* = biologic dose needed to compensate for repopulation, which was assumed to be 0.6 Gy; *T* = total treatment time in days; and *T*_*delay*_ = time until onset of repopulation, assumed to be 21 days. For regimens lasting less than 21 days, *k* was set to zero (17).

### Statistical analyses

Analyses were based on median survival times (18), extracted from trial publications for each arm. If not reported, they were derived from survivor function graphs or 1-year survival. For each randomized comparison, a median survival ratio was calculated by dividing median survival in the higher-dose arm by median survival in the lower-dose arm. For trials with more than 2 study arms, separate median survival ratios compared with the lowest-dose arm were calculated for every other trial arm, resulting in separate treatment comparisons. Confidence intervals and significance tests were based on standard errors (19). All analyses were conducted in Stata version 13.0 (20). Further methodologic details are in Figure E2 (available online at www.redjournal.org).

## Results

**Fig. 1:**
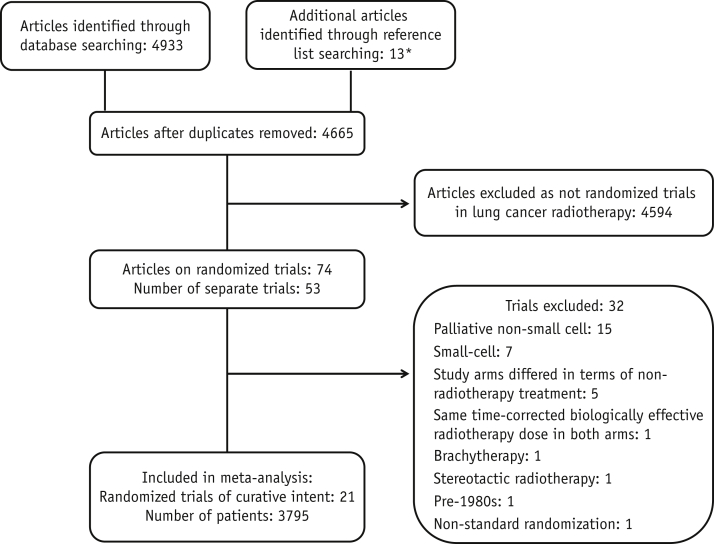
Trial identification and selection. ^∗^Reference lists in publications for all trials included in the study and for other meta-analyses of radiation therapy in lung cancer were searched to identify additional publications of trials missed in the search.

Twenty-one trials with 3795 patients were included in the meta-analysis (Fig. 1) 6, 21, 22, 23, 24, 25, 26, 27, 28, 29, 30, 31, 32, 33, 34, 35, 36, 37, 38, 39, 40. Eight trials were conducted in China, 7 in North America, 4 in Europe, 1 in South Asia, and 1 in Australia (Table 1). (For further details of study characteristics and treatments, see Tables E1 and E2; available online at www.redjournal.org.)

The number of patients in each trial ranged from 30 to 563, the years of randomization from 1982 to 2011, and the average age at randomization from 48 to 66 years (Table E1; available online at www.redjournal.org). In the 18 trials reporting proportions of patients by cancer stage, 13 reported that at least 94% of patients had stage III disease, and 5 reported that between 55% and 89% had stage III disease. Two trials had multiple arms, resulting in 25 randomized radiation therapy dose comparisons in 21 trials (Table 1). The EQD2T doses within trial arms ranged from 36.4 Gy to 80.8 Gy (Table E2; available online at www.redjournal.org), and EQDT2 increased with calendar year (*P*=.04) (Fig. E3; available online at www.redjournal.org). The dose difference between trial arms ranged from 1.1 Gy to 27.2 Gy (Table 1), and in 12 of the randomized comparisons, dose escalation was achieved by hyperfractionation. Chemotherapy was given in 7 trials (2 sequential, 5 concurrent) and was not given in 18. The median survival times ranged from 6.3 to 29.9 months.

### Effect of chemotherapy on dose escalation

**Fig. 2:**
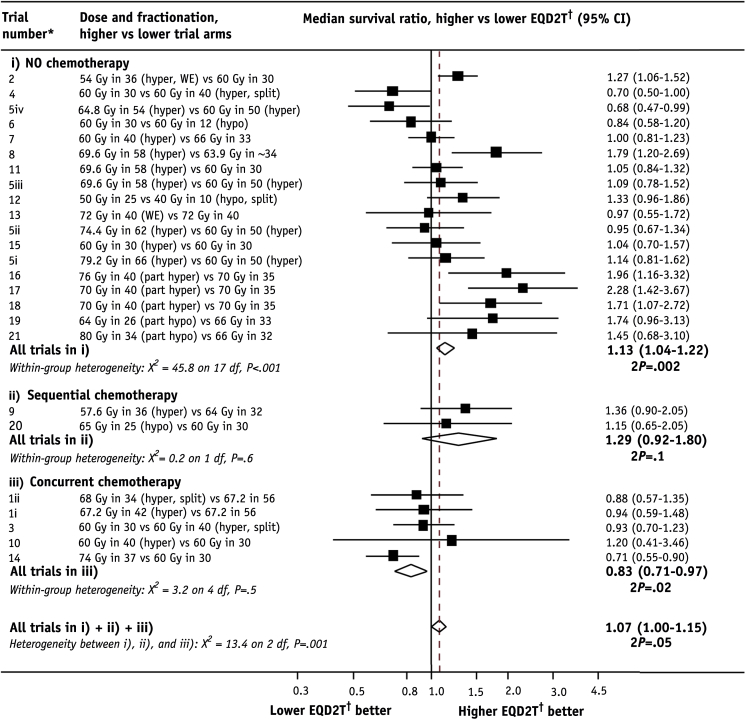
Median survival ratios, higher versus lower corrected radiation therapy dose (EQD2T), according to whether radiation therapy was given without chemotherapy, with sequential chemoradiation therapy, or with concurrent chemoradiation therapy. *Abbreviations:* hyper = hyperfractionated (>1 fraction per day); hypo = hypofractionated (>2 Gy per fraction); part hyper = partially hyperfractionated; part hypo = partially hypofractionated; split = split-course radiation therapy, minimum 10-day gap; WE = including weekends. ^∗^Studies are ordered within groups by ascending EQD2T difference between trial arms. ^†^EQD2T is time-corrected equivalent dose in 2-Gy fractions.

The median overall survival ratio, higher versus lower EQD2T, pooled across all randomized dose comparisons, was 1.07 (95% CI 1.00-1.15, *P*=.05) (Fig. 2). When these comparisons were grouped according to whether the protocol specified no chemotherapy, sequential chemotherapy, or concurrent chemotherapy the heterogeneity between these groups was highly significant (*P*=.001). The median survival ratios were 1.13 (95% CI 1.04-1.22, *P*=.002) without chemotherapy, 1.29 (95% CI 0.92-1.80, *P*=.1) with sequential chemotherapy, and 0.83 (95% CI 0.71-0.97, *P*=.02) with concurrent chemotherapy. Hence, radiation therapy dose escalation led to significantly better survival for comparisons without chemotherapy but to significantly poorer survival in comparisons with concurrent chemotherapy.

For randomized comparisons that included concurrent chemotherapy, there was no significant heterogeneity between the median survival ratios (*P*=.5), nor was there any trend with increasing EQD2T difference between arms (*P*=.1). There were only 2 randomized comparisons with sequential chemotherapy, including 193 patients, so the confidence intervals were wide.

### Radiation therapy dose escalation without chemotherapy

**Fig. 3:**
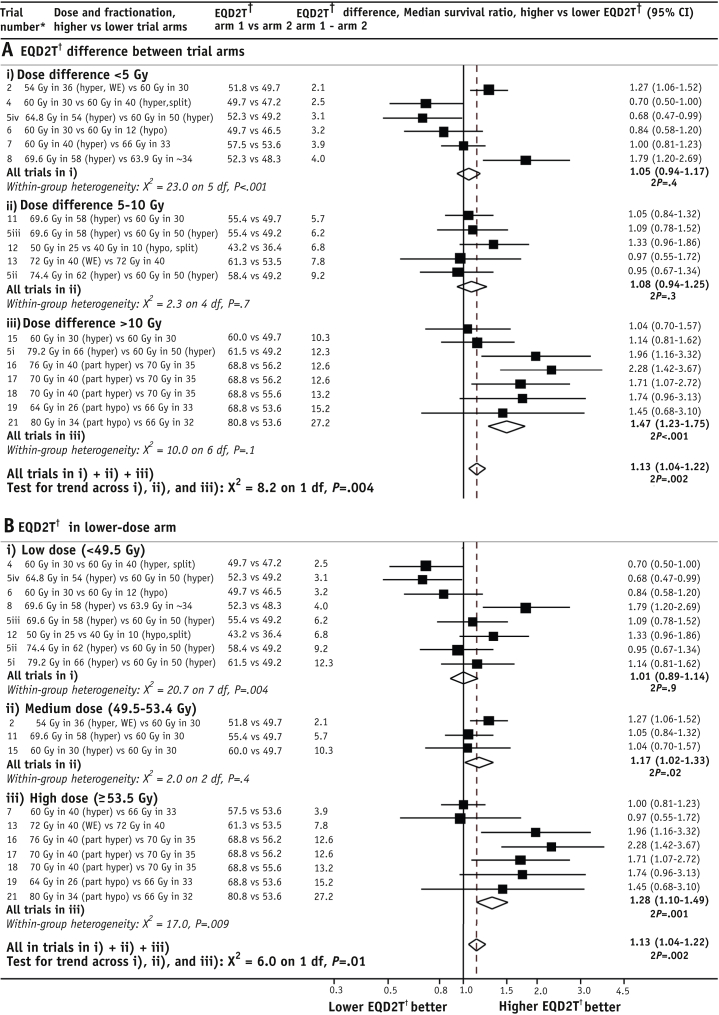
Median survival ratios, higher versus lower corrected radiation therapy dose (EQD2T). (A) Categorized by EQD2T difference between arms. (B) Categorized by EQD2T in the lowest-dose arm. Trials with chemotherapy excluded. *Abbreviations:* hyper = hyperfractionated (>1 fraction per day); hypo = hypofractionated (>2 Gy per fraction); part hyper = partially hyperfractionated; part hypo = partially hypofractionated; split = split-course radiation therapy, minimum 10-day gap; WE = including weekends. ^∗^Studies are ordered within groups by ascending EQD2T difference between trial arms. ^†^EQD2T is time-corrected equivalent dose in 2-Gy fractions.

**Fig. 4:**
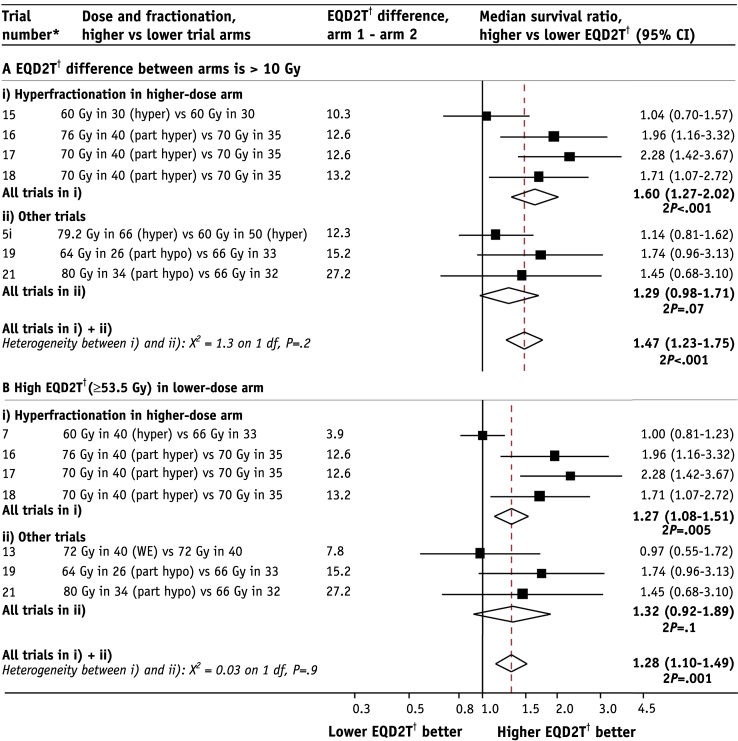
Median survival ratios, higher versus lower corrected radiation therapy dose (EQD2T), according to whether higher dose was achieved by hyperfractionation or by other means. (A) Trials with EQD2T >10 Gy dose difference between arms (ie, group iii in Fig. 3A). (B) Trials with EQD2T in the lower-dose arm ≥53.5 Gy (ie, group iii in Fig. 3B). Trials with chemotherapy excluded. *Abbreviations:* hyper = hyperfractionated (>1 fraction per day); hypo = hypofractionated (>2 Gy per fraction); part hyper = partially hyperfractionated; part hypo = partially hypofractionated; split = split-course radiation therapy, minimum 10-day gap; WE = including weekends. ^∗^Studies are ordered within groups by ascending EQD2T difference between trial arms. ^†^EQD2T is time-corrected equivalent dose in 2-Gy fractions.

For the 18 radiation therapy–only comparisons, the median survival ratio, higher versus lower radiation therapy dose, was 1.13 (95% CI 1.04-1.22) (Fig. 2). When these comparisons were categorized according to dose difference between trial arms, the pooled median survival ratio for EQD2T differences of <5 Gy was 1.05 (95% CI 0.94-1.17, *P*=.4), whereas for EQD2T differences of 5 to 10 Gy it was 1.08 (95% CI 0.94-1.25, *P*=.3), and for EQD2T differences of >10 Gy, it was 1.47 (95% CI 1.23-1.75, *P*<.001) (Fig. 3A). The increasing trend in median survival ratios across these 3 groups was highly statistically significant (*P* for trend=.004), providing strong evidence that without chemotherapy, survival increases with increasing EQD2T. When the dose comparisons with EQD2T differences of ≥10 Gy between trial arms were further divided into trials in which hyperfractionation was used to escalate dose versus other trials, the median survival ratios did not differ significantly (1.60, 95% CI 1.27-2.02, and 1.29, 95% CI 0.98-1.71, *P* for difference=.2) (Fig. 4A).

When the 18 radiation therapy–only comparisons were grouped according to dose in the lower-dose arm into 3 approximately equal-sized groups (Fig. 3B), the median survival ratio, higher versus lower dose, was 1.01 (95% CI 0.89-1.14, *P*=.9) for comparisons where the EQD2T in the lower-dose arm was <49.5 Gy, whereas for comparisons in which it was 49.5 to 53.4 Gy, the median survival ratio was 1.17 (95% CI 1.02-1.33, *P*=.02), and for comparisons in which it was ≥53.5 Gy, it was 1.28 (95% CI 1.10-1.49, *P*<.002). So, surprisingly, the median survival ratio increased progressively with increasing EQD2T in the lower-dose arm, with an increasing trend across the 3 groups (*P* for trend=.01). Even for trials with EQD2T ≥53.5 Gy in the lower-dose arm, further dose escalation provided an additional improvement in survival. For the 5 trials in which the dose in the lower-dose arm was EQD2T ≥53.5 Gy and where the dose difference was EQD2T >10 Gy, the median survival ratio was 1.87 (95% CI 1.47-2.38, *P*<.001). When comparisons with EQD2T ≥53.5 Gy in the lower-dose arm were categorized into trials in which hyperfractionation was used to escalate dose versus other trials, the median survival ratio for the 2 groups was similar (1.27, 95% CI 1.08-1.51, and 1.32, 95% CI 0.92-1.89, *P* for difference=.9) (Fig. 4B).

### Heterogeneity between trials and exploratory analyses

**Fig. 5:**
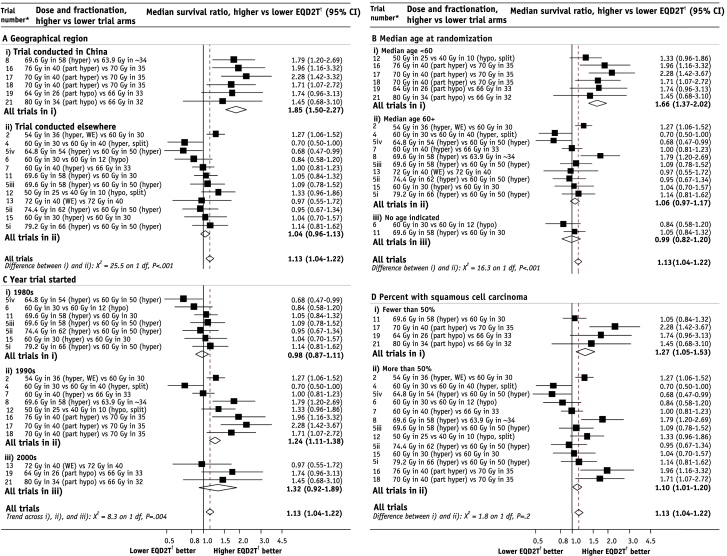
Median survival ratios, higher versus lower corrected radiation therapy dose (EQD2T). (A) According to geographic region. (B) According to median age at randomization. (C) According to year trial started. (D) According to percentage of patients with squamous cell carcinoma. Trials with chemotherapy excluded. *Abbreviations:* hyper = hyperfractionated (>1 fraction per day); hypo = hypofractionated (>2 Gy per fraction); part hyper = partially hyperfractionated; part hypo = partially hypofractionated; split = split-course radiation therapy, minimum 10-day gap; WE = including weekends. ^∗^Studies are ordered within groups by ascending EQD2T difference between trial arms. ^†^EQD2T is time-corrected equivalent dose in 2-Gy fractions.

The median survival ratios for the 18 radiation therapy–only dose comparisons varied substantially, from 0.68 (95% CI 0.47-0.99) to 2.28 (95% CI 1.42-3.67) (*P* for heterogeneity<.001) (Fig. 2). The difference in EQD2T between trial arms did not account for all the excess heterogeneity, so exploratory analyses were conducted to examine the associations between median survival ratios and other available factors (Fig. 5). The median survival ratio, higher versus lower EQD2T, was higher for trials conducted in China than for trials conducted elsewhere (China: 1.85, 95% CI 1.50-2.27; elsewhere: 1.04, 95% CI 0.96-1.13, *P* for difference <.001) (Fig. 5A), for trials with lower median age (<60 years: 1.66, 95% CI 1.37-2.02, 60+ years: 1.06 95% CI 0.97-1.17, *P* for difference <.001) (Fig. 5B), and for trials starting more recently (1980s: 0.98, 95% CI 0.87-1.11, 1990s: 1.24, 95% CI 1.11-1.38, 2000s: 1.32, 95% CI 0.92-1.89, *P* for trend=.004) (Fig. 5C). By contrast, when the trials were grouped according to whether most patients had squamous cell carcinoma (SCC), no significant difference was found (*P*=.2) (Fig. 5D). Geographic region, age, and year trial started were correlated, whereas percentage of patients with SCC was not highly correlated with any other factor (Table E3; available online at www.redjournal.org). An analysis adjusting for all 4 factors showed the strongest association between geographic region and median survival ratio (*P*=.008), with all other factors statistically nonsignificant (Table E4; available online at www.redjournal.org).

## Discussion

This large meta-analysis including 3795 patients in 25 treatment comparisons has, for the first time, brought together all post-1980 randomized evidence comparing different curative-intent radiation therapy regimens in NSCLC. To eliminate the confounding effects of other treatments, we considered only trials in which other protocol treatments were identical in both arms. We focused on median survival ratios in relation to corrected dose (ie, time-corrected equivalent dose, EQD2T) differences between trial arms and whether the radiation therapy was given alone or with chemotherapy.

### Radiation therapy without chemotherapy

In 18 trials where no protocol chemotherapy was administered, radiation therapy dose escalation improved overall survival (median survival ratio: 1.13, 95% CI 1.04-1.22), corresponding to a survival gain of approximately 2 months for patients in these trials. The survival improvement increased progressively as the difference between EQD2T in the 2 trial arms increased and, in trials where the difference was EQD2T >10 Gy, the median survival ratio was 1.47 (95% CI 1.23-1.75). Remarkably, even for trials where the dose in the lower-dose arm was high (EQD2T ≥53.5 Gy), further dose escalation provided an additional improvement in survival (median survival ratio: 1.28, 95% CI 1.10-1.49) (Fig. 3B). In trials with EQD2T ≥53.5 Gy in the lower-dose arm where the dose difference exceeded EQD2T 10 Gy, the median survival ratio was 1.87 (95% CI 1.47-2.38), and for these patients the survival gain was approximately 1 year.

The commonest method of altering dose and fractionation in these trials was hyperfractionation. Two previous meta-analyses of different radiation therapy regimens focused on comparing hyperfractionation or acceleration with conventional radiation, rather than on corrected dose 8, 10. In our study, dose escalation of EQD2T >10 Gy showed a similar improvement in survival regardless of whether it was achieved by hyperfractionation or by other means, suggesting that dose escalation may improve survival regardless of the method used.

Survival improvement from radiation therapy dose escalation was associated with 3 other factors: younger age, recent trial start date, and whether the trial was conducted in China. The strongest association was for trials conducted in China. Nothing in the trial publications indicated that the Chinese trials differed systematically from other trials, and inasmuch as the 3 factors were highly correlated, the geographic association may not be causal. By contrast, it would not be surprising if radiation therapy dose escalation had a greater benefit in younger patients, who may be better able to tolerate radiation therapy than older patients, and thus may receive the full prescribed dose. Younger patients may also face a lower risk of death resulting from toxicity than older patients, and have lower competing risks of death from other causes. Survival improvement was also greater in more recent trials. This could be due in part to increased prescribed tumor doses and improved radiation therapy techniques. There was no significant association between dose escalation and the percentage of patients with SCC, but for most trials, the percentage of patients with SCC was between 40% and 60%, so there was little variation in this factor.

### Radiation therapy with chemotherapy

In trials with concurrent chemotherapy, higher EQD2T led to poorer overall survival, suggesting that the risks of increasing radiation therapy dose outweigh the benefits when concurrent chemotherapy is given (Fig. 2). There was no evidence of heterogeneity in the median survival ratios for trials in this group, but the pooled result was dominated by the RTOG 0617 trial (number 14), in which 207 patients randomized to EQD2T 58.8 Gy (74 Gy in 37 fractions) had higher overall mortality than did 217 patients randomized to EQD2T 49.7 Gy (60 Gy in 30 fractions) (HR, higher vs lower dose, 1.38, 95% CI 1.09-1.76, *P*=.004); the higher-dose group also had more grade 3 or worse esophageal toxicity (21% vs 7%, *P*<.001) (6). The results of this trial are influencing radiation dose escalation in chemoradiation therapy, as reflected in the 2015 American Society for Radiation Oncology guidelines (7); however, there are arguments why this single trial should not stop further exploration of dose escalation in chemoradiation therapy (41). One of these is that there was reduced protocol adherence in the higher-EQD2T arm (higher dose: 153/207 [74%], lower dose: 180/217 [83%], *P*=.02), which may explain the nonsignificantly increased local failure in the higher-dose group (HR 1.26, 95% CI 0.93-1.71, *P*=.1) (6).

Only 2 trials compared different radiation therapy regimens with the same sequential chemotherapy in both arms. Hence, information on dose escalation with sequential chemotherapy is limited.

### Radiation therapy, chemotherapy, and toxicity

The probability of sterilizing a tumor increases with increasing tumor radiation dose (42). However, lung cancer radiation therapy inevitably delivers dose to normal lung and adjacent organs that may cause pneumonitis, esophagitis, heart disease, and lung fibrosis 43, 44, 45. Adding chemotherapy to radiation therapy further increases toxicity, including myelosuppression, esophagitis (up to 6 times higher with concurrent chemotherapy), pneumonitis, and treatment-related death (46). Increasing the irradiated volume alongside concurrent chemotherapy can also increase toxicity (47). Toxicity is thus a primary constraint in increasing radiation and chemotherapy dose in NSCLC treatment, especially because patients often have multiple comorbidities. In this study, it was not possible to analyze the effects of toxicity on median survival because of the variability in toxicity reporting across trials in terms of type, severity, and time point.

Modern radiation techniques are being developed to reduce toxicity. They include intensity modulated radiation therapy, proton beam therapy, and personalized isotoxic radiation therapy, in which patients receive maximal radiation therapy dose based on normal tissue constraints and their individual tumor size and location (48). In future, these efforts to reduce toxicity may facilitate radiation therapy dose escalation, even when chemotherapy is also given.

### Strengths and limitations

Our meta-analysis has several strengths. It is larger and has more power than previously published meta-analyses of trials of curative-intent radiation therapy for lung cancer 8, 9, 10. We have, for the first time, examined trials both according to the corrected radiation therapy dose and to whether chemotherapy was delivered. Most importantly, we have included total treatment time in our dose calculation (EQD2T rather than EQD2). Omitting the time factor would have resulted in substantially different results because it would have reversed the higher and lower dose arms for 7 trials (numbers 2, 6, 7, 9, 10, 17, and 18), and for 2 further trials (numbers 13 and 15) the corrected doses would have been identical in the 2 arms. This is because in 7 of these trials, 1 of the arms was hyperfractionated (more dose intense) whereas the other was conventionally fractionated.

The main limitation of our study is a lack of individual patient data. Effects seen at the aggregate level may be weaker or stronger than those examined at the individual level. This also prevented us from considering survival at 2 years, by which time most patients with occult distant metastases when irradiated would have died. We also could not conduct analyses of deaths resulting from lung cancer versus other causes. Another limitation is that the calculation of EQD2T involves radiobiologic assumptions about the way in which radiation kills tumor cells, including values of parameters. For example, in the CHART trial (number 2), 67% of patients in the higher-dose arm versus 34% in the lower-dose arm experienced severe esophagitis, although the EQD2T difference to the tumor was calculated to be only 2.1 Gy (49).

## Conclusions

In conclusion, survival in locally advanced NSCLC patients has seen little improvement over 40 years (2), so any evidence of improvement in survival is clinically important. Our study shows that when radiation therapy is given without chemotherapy, escalating radiation therapy dose leads to improved survival. This suggests that the optimal radiation dose has yet to be reached, and it provides support for further trials of dose escalation, especially with modern radiation therapy techniques that lower toxicity. When chemotherapy is given with radiation therapy, the ability to achieve a cure has, until now, been limited by toxicity, especially when it is given concurrently. Therefore, it may be that the optimal radiation dose for chemoradiation therapy has also not yet been reached in the context of new advances, such as personalized isotoxic dose escalation, and in the continued search for the optimal concurrent chemotherapy regimen. Our study therefore also provides support for consideration of further trials in radiation dose escalation of chemoradiation therapy for locally advanced NSCLC.

## Figures and Tables

**Table 1:** Descriptive summary of trials included in meta-analysis, by ascending EQD2T difference between trial arms∗

Trial no.	Study, y (reference)	Years of randomization	Country	No. of patients	EQD2T in each trial arm (Gy)	EQD2T difference between trial arms (Gy)	Chemotherapy
1	Zhan et al, 2007 (39)	2000-2005	China	159	A: 56.2, B: 54.9, C: 53.8	1.1, 2.4†	Concurrent
2	Saunders et al, 1999 (23)	1990-1995	UK, Germany, Sweden	563	A: 51.8, B: 49.7	2.1	None
3	Schild et al, 2002 (24)	1994-1999	USA	234	A: 49.7, B: 47.2	2.5	Concurrent
4	Bonner et al, 1998 (25)	1992-1993	USA	67	A: 49.7, B: 47.2	2.5	None
5	Cox et al, 1990 (26)	1983-1987	USA	516	A: 61.5, B: 58.4, C: 55.4, D: 52.3, E: 49.2	3.1, 6.2, 9.2, 12.3‡	None
6	Slawson et al, 1988 (27)	1982-1986	USA	120	A: 49.7, B: 46.5	3.2	None
7	Baumann et al, 2011 (21)	1997-2005	Germany, Poland, Czech Republic	406	A: 57.5, B: 53.6	3.9	None
8	Fu et al, 1994 (28)	1990-1992	China	105	A: 52.3, B: 48.3	4.0	None
9	Belani et al, 2005 (29)	1998-2001	USA	119	A: 55.7, B: 51.6	4.1	Sequential
10	Sapkota et al, 2013 (30)	Not specified	India, Nepal	30	A: 54.2, B: 49.7	4.5	Concurrent
11	Sause et al, 2000 (31)	1989-1992	USA, Canada	301	A: 55.4, B: 49.7	5.7	None
12	Reinfuss et al, 1999 (32)	1992-1996	Poland	160	A: 43.2, B: 36.4	6.8	None
13	Zajusz et al, 2006 (36)	2001-2006	Poland	53	A: 61.3, B: 53.5	7.8	None
14	Bradley et al, 2015 (6)	2007-2011	USA, Canada	424	A: 58.8, B: 49.7	9.1	Concurrent
15	Ball et al, 1999 (22)	1989-1995	Australia	99	A: 60.0, B: 49.7	10.3	None
16	Zhu et al, 2000 (40)	1993-1996	China	70	A: 68.8, B: 56.2	12.6	None
17	Cheng W et al, 2007 (38)	1999-2002	China	81	A: 68.8, B: 56.2	12.6	None
18	Cheng J et al, 2004 (37)	1995-1998	China	74	A: 68.8, B: 55.6	13.2	None
19	Wang et al, 2005 (33)	2001-2003	China	86	A: 68.8, B: 53.6	15.2	None
20	Yu et al, 2014 (35)	2009-2011	China	60	A: 68.8, B: 53.2	25.6	Sequential
21	Wang et al, 2008 (34)	2004-2006	China	68	A: 80.8, B: 53.6	27.2	None

∗ EQD2T is calculated in terms of 2-Gy biologically equivalent dose per fraction, corrected for total treatment time. Study arms are presented in order of ascending difference in EQD2T between trial arms; if there were multiple arms, study number was assigned based on the smallest dose difference.

† Three-arm study. Each of the higher-dose arms was separately compared with the lowest-dose arm as the baseline. Arm B versus C: 1.1-Gy difference. Arm A versus C: 2.4-Gy difference.

‡ Five-arm study. Each of the higher-dose arms was separately compared with the lowest-dose arm as the baseline. Arm D versus E: 3.1-Gy difference. Arm C versus E: 6.2-Gy difference. Arm B versus E: 9.2-Gy difference. Arm A versus E: 12.3-Gy difference.
