# Supplementary material for: Dose and Fractionation in Radiation Therapy of Curative Intent for Non-Small Cell Lung Cancer: Meta-Analysis of Randomized Trials
Source: Int J Radiat Oncol Biol Phys. 2016 Nov 15;96(4):736–47. doi: 10.1016/j.ijrobp.2016.07.022 (PMC5082441; doi:10.1016/j.ijrobp.2016.07.022)
Supplement: Supplementary Material [file mmc1.pdf]

Dose and fractionation in radiotherapy of curative intent for non-small-cell lung cancer: Meta-analysis of randomized trials

Johanna Ramroth, David J. Cutter, Sarah C. Darby, Geoff S. Higgins, Paul McGale, Mike Partridge, Carolyn W. Taylor

Appendix

Figure E1. Terms used in Embase to search for eligible trials from 1 January 1974 to 28 April, 2015.

Figure E2. Additional methodological details.

Table E1. Trials included in meta-analysis, ordered according to EQD2T radiotherapy dose difference between trial arms: patient characteristics, outcomes and any excluded trial arms.

Table E2. Trials included in meta-analysis, ordered according to EQD2T radiotherapy dose difference between trial arms: protocol treatment information, radiotherapy and chemotherapy.

Figure E3. EQD2T radiotherapy dose in individual arms of trials comparing different radiotherapy regimens, with or without chemotherapy, by year trial started.

Table E3. Correlation of geographical region, average age at trial entry, year trial started, and percent of patients with squamous cell carcinoma.

Table E4. Variation in pooled median survival ratio, higher versus lower EQD2T dose, by average age at trial entry, geographical region, year trial started, and percent of patients with squamous cell carcinoma.

Figure E4. Funnel plot of median survival ratios and standard errors of randomized treatment comparisons in which radiotherapy is given without chemotherapy.

Figure E1. Terms used in Embase to search for eligible trials from 1 January 1974 to 28 April, 2015.

An initial search was conducted (A below). This search was updated (B below) using narrower search terms. A sensitivity analysis was conducted in the older search using the narrower search terms to ensure that the new search would include all articles of interest without excluding any. The original searches were conducted using the full Embase database, which starts 1 January, 1974. However, it was found that a substantial number of trials published prior to 1980 used old radiotherapy methods, such as Cobalt. It was therefore decided, prior to conducting any analyses, only to include trials starting in 1980 or later.

A. In search conducted up to 12 September, 2013:

1. exp radiation dose fractionation/
2. fraction*.mp. [mp=title, abstract, subject headings, heading word, drug trade name, original title, device manufacturer, drug manufacturer, device trade name, keyword]
3. (hypofraction* or hyperfraction*).mp. [mp=title, abstract, subject headings, heading word, drug trade name, original title, device manufacturer, drug manufacturer, device trade name, keyword]
4. "accelerat* radiotherap*".mp. [mp=title, abstract, subject headings, heading word, drug trade name, original title, device manufacturer, drug manufacturer, device trade name, keyword]
5. (radiotherapy adj5 (regimen* or schedule*)).mp. [mp=title, abstract, subject headings, heading word, drug trade name, original title, device manufacturer, drug manufacturer, device trade name, keyword]
6. human/
7. letter/
8. editorial/
9. phase 1 clinical trial/
10. 7 or 8 or 9
11. exp *lung cancer/ or exp *lung tumor/ or exp *lung carcinoma/
12. exp *pleura cancer/ or exp *pleura tumor/
13. exp *bronchus cancer/ or exp *bronchus tumor/
14. (bronch* adj5 (neoplas* or carcinom* or cancer* or tumor* or tumour*)).m_titl.
15. (lung adj5 (neoplas* or carcinom* or cancer* or tumor* or tumour*)).m_titl.
16. (pleura* adj5 (neoplas* or carcinom* or cancer* or tumor* or tumour*)).m_titl.
17. 11 or 12 or 13 or 14 or 15 or 16
18. 1 or 2 or 3 or 4 or 5
19. 6 and 17 and 18
20. 19 not 9

B. In search update from 12 September, 2013 to April 28, 2015:

1. exp radiation dose fractionation/
2. fraction*.mp. [mp=title, abstract, subject headings, heading word, drug trade name, original title, device manufacturer, drug manufacturer, device trade name, keyword]
3. (hypofraction* or hyperfraction*).mp. [mp=title, abstract, subject headings, heading word, drug trade name, original title, device manufacturer, drug manufacturer, device trade name, keyword]
4. "accelerat* radiotherap*".mp. [mp=title, abstract, subject headings, heading word, drug trade name, original title, device manufacturer, drug manufacturer, device trade name, keyword]
5. human/
6. letter/
7. editorial/
8. phase 1 clinical trial/
9. 6 or 7 or 8
10. exp *lung cancer/ or exp *lung tumor/ or exp *lung carcinoma/
11. exp *pleura cancer/ or exp *pleura tumor/
12. exp *bronchus cancer/ or exp *bronchus tumor/
13. (bronch* adj5 (neoplas* or carcinom* or cancer* or tumor* or tumour*)).mp. [mp=title, abstract, subject headings, heading word, drug trade name, original title, device manufacturer, drug manufacturer, device trade name, keyword]
14. (lung adj5 (neoplas* or carcinom* or cancer* or tumor* or tumour*)).mp. [mp=title, abstract, subject headings, heading word, drug trade name, original title, device manufacturer, drug manufacturer, device trade name, keyword]
15. (pleura* adj5 (neoplas* or carcinom* or cancer* or tumor* or tumour*)).mp. [mp=title, abstract, subject headings, heading word, drug trade name, original title, device manufacturer, drug manufacturer, device trade name, keyword]
16. 10 or 11 or 12 or 13 or 14 or 15
17. (bronch* adj5 (neoplas* or carcinom* or cancer* or tumor* or tumour*)).m_titl.
18. (lung adj5 (neoplas* or carcinom* or cancer* or tumor* or tumour*)).m_titl.
19. (pleura* adj5 (neoplas* or carcinom* or cancer* or tumor* or tumour*)).m_titl.
20. 10 or 11 or 12 or 17 or 18 or 19
21. (radiotherapy adj5 (regimen* or schedule*)).mp. [mp=title, abstract, subject headings, heading word, drug trade name, original title, device manufacturer, drug manufacturer, device trade name, keyword]
22. 1 or 2 or 3 or 4 or 21
23. 5 and 16 and 22
24. 23 not 9
25. 5 and 20 and 22
26. 25 not 9
27. "accelerat* adj3 radiotherap*".mp.
28. (accelerat* adj3 radiotherap*).mp.
29. 1 or 2 or 3 or 28 or 21

continued on next page

continued from previous page

30. 5 and 20 and 29

31. 30 not 9

32. exp radiation dose fractionation/

33. fraction*.mp. [mp=title, abstract, subject headings, heading word, drug trade name, original title, device manufacturer, drug manufacturer, device trade name, keyword]

34. (hypofraction* or hyperfraction*).mp. [mp=title, abstract, subject headings, heading word, drug trade name, original title, device manufacturer, drug manufacturer, device trade name, keyword]

35. "accelerat* radiotherap*".mp. [mp=title, abstract, subject headings, heading word, drug trade name, original title, device manufacturer, drug manufacturer, device trade name, keyword]

36. human/

37. letter/

38. editorial/

39. phase 1 clinical trial/

40. 37 or 38 or 39

41. exp *lung cancer/ or exp *lung tumor/ or exp *lung carcinoma/

42. exp *pleura cancer/ or exp *pleura tumor/

43. exp *bronchus cancer/ or exp *bronchus tumor/

44. (bronch* adj5 (neoplas* or carcinom* or cancer* or tumor* or tumour*)).mp. [mp=title, abstract, subject headings, heading word, drug trade name, original title, device manufacturer, drug manufacturer, device trade name, keyword]

45. (lung adj5 (neoplas* or carcinom* or cancer* or tumor* or tumour*)).mp. [mp=title, abstract, subject headings, heading word, drug trade name, original title, device manufacturer, drug manufacturer, device trade name, keyword]

46. (pleura* adj5 (neoplas* or carcinom* or cancer* or tumor* or tumour*)).mp. [mp=title, abstract, subject headings, heading word, drug trade name, original title, device manufacturer, drug manufacturer, device trade name, keyword]

47. 41 or 42 or 43 or 44 or 45 or 46

48. (bronch* adj5 (neoplas* or carcinom* or cancer* or tumor* or tumour*)).m_titl.

49. (lung adj5 (neoplas* or carcinom* or cancer* or tumor* or tumour*)).m_titl.

50. (pleura* adj5 (neoplas* or carcinom* or cancer* or tumor* or tumour*)).m_titl.

51. 41 or 42 or 43 or 48 or 49 or 50

52. (radiotherapy adj5 (regimen* or schedule*)).mp. [mp=title, abstract, subject headings, heading word, drug trade name, original title, device manufacturer, drug manufacturer, device trade name, keyword]

53. 32 or 33 or 34 or 35 or 52

54. 36 and 47 and 53

55. 54 not 40

56. 36 and 51 and 53

57. 56 not 40

58. "accelerat* adj3 radiotherap*".mp.

59. (accelerat* adj3 radiotherap*).mp.

continued on next page

continued from previous page

60. 32 or 33 or 34 or 59 or 52

61. 36 and 51 and 60

62. 61 not 40

63. 62

64. limit 63 to yr="2013 -Current"

65. (kinase or centrifugal or retinoic or ascorbate or antigen or c-myc or keratin or gefitinib or telomerase or "in vitro" or "cytometry" or "cytometric" or p53 or p50 or p54 or p73 or p63 or HtrA3 or KRAS or K-RAS or thyrosinase or allele or LLC1).m_titl.

66. 64 not 65

67. (lipid or plasminogen or proteolysis or mutation or biogenesis or heterozygosity or monoclonal or apoptosome or kinetic or inhibitor or tenascin or isoenzyme or enzyme or CDKN2 Phenethyl or immunoreactivity or epigenetic or methylation or entropy).m_titl.

68. (beta-carotene or retinoid or annexin or herbal or proteomics or centrosome or monoclonal or clonal or "effector cell" or immunostaining or lectin or lymphokine or Dendritic or immunotherapy or macrophage or RNA or receptor or "cell line" or "cell lines" or xenograft).m_titl.

69. 65 or 67 or 68

70. 64 not 69

71. small cell lung cancer/ or lung small cell cancer/

72. 70 not 71

73. brain metastasis/ or skull irradiation/

74. 72 not 73

75. ("prophylactic cranial irradiation" or PCI).m_titl.

76. 74 not 75

77. patient*.ab.

78. 76 and 77

79. random*.ab.

80. 78 and 79

Figure E2. Additional methodological details

The Preferred Reporting Items for Systematic Reviews and Meta-Analyses (PRISMA) guidelines specify that a systematic review should consider possible risks of bias, both within studies and between them (1). To this end, we provide details regarding trial identification, the outcome measure, small study effects, and heterogeneity between trials. We also provide some other methodological details.

Method of trial identification

The search criteria were reviewed by two authors (CT and SD) and a librarian. Abstracts were screened by one author (JR) and included trials were reviewed by three (JR, CT, and SD). Decisions on the inclusion of trials of SCLC versus NSCLC, palliative versus curative intent radiotherapy, cut-off year (1980), methods of randomization, and language were made by JR, CT, and SD. Trials for which methods of randomization were not indicated in the publication were included, but one trial stating the use of hospital numbers to randomize was excluded (2). Eligible trials for which publications were not in English (German, French, and Chinese) were translated and included, and three further articles (Russian and Japanese) were reviewed and deemed ineligible. We successfully contacted authors of three Chinese articles in order to obtain manuscripts not otherwise available. We contacted authors of a Polish study for which only an abstract was available, and obtained information about numbers of deaths and median survival in each trial arm.

Outcome measure

Median survival was selected as our outcome measure as it has good statistical properties (3) and it was the measure most frequently available in eligible trials. Five trials (study numbers 1, 8, 16, 17, 18) did not provide information on median survival and had no Kaplan-Meier plot, so median survival time was estimated from 1-year survival assuming that the survival times had an exponential distribution. . In the three trials in which median survival had to be read from plots (study numbers 5, 7, 15), these were magnified and a ruler was used.

The standard error (SE) for the log of the median survival ratio in each trial was estimated as follows:

$$SE_{log\_median\_survival\_ratio} = \sqrt{\frac{1}{D_1} + \frac{1}{D_2}},$$

where D_1 is the number of patients who died in arm 1 and D_2 the number who died in arm 2 (4).

The number of patients who had died in each study arm was obtained as follows in the 21 trials: in four trials, the numbers were stated in the text and in one it was obtained via personal communication from study investigators. In one further study, the numbers were estimated from median survival time or median follow-up time; and in the remaining 15 studies, numbers died were estimated from the Kaplan Meier plot or the proportion surviving at the longest reported period (eg. at 3 or 5 years). If numbers were estimated from a Kaplan Meier plot, the proportion died was assumed to be the proportion not surviving with no censoring due to loss to follow-up.

Use of the median survival could lead to some dilution of effect, as some patients with unidentified metastatic disease at the outset may still be alive at the median survival point, whereas most of these patients would have died by a later point, for example at two years. Unfortunately, two-year survival could not be used, because obtaining a standard error for two-year survival was not possible, as rates of death in each trial arm were not available.

Statistical analyses

We used fixed-effects meta-analysis. Three types of statistical test formed the basis of results presented in forest plots. Within-group heterogeneity was calculated in order to assess whether variability between studies was greater than due to the play of chance. Between-group heterogeneity was assessed in order to determine whether subgroups were statistically different from one another. In order to assess trends, either across studies ordered within a group, or between ordered groups, chi-squared tests for trend were conducted.

The test for heterogeneity we used in the various meta-analyses was a weighted chi-squared test (Cochran's Q chi-squared test statistic) as follows:

$$Q = \sum_i \left[\left(\frac{1}{v_i}\right) * \left(\mathrm{Effect}_i - \mathrm{Effect}_{\mathrm{pooled}}\right)^2\right],$$

where Q is distributed on N-1 degrees of freedom, Effect_i is the log median survival ratio of a given study, $\mathrm{Effect}_{\mathrm{pooled}}$ is the overall pooled log median survival ratio, and v_i is the variance of the log median survival ratio of a given study (the square of the standard error given above).

Small study effects

A funnel plot was generated in order to assess whether there may be small study effects, including publication bias (Fig. E4). Funnel plots scatter study effects by size of the study, such that bigger studies' effect sizes should be closer to the overall effect size, while smaller studies will vary more. However, regardless of study size, their effects should scatter symmetrically about the overall effect determined in the meta-analysis (5). Small studies are sometimes less likely to be published than big studies if they do not show a statistically significant effect. There may also be systematic reasons why smaller studies show bigger effects (5). Either way, small study effects are more prone to result in asymmetry around the effect line. In our meta-analysis, however, there was no visible asymmetry among the smaller trials (Fig. E4). Egger's test of no linear association between the treatment effect and its standard error was performed in order to assess asymmetry in the plot (p=0.3) (6).

Sources of heterogeneity

There was evidence of significant heterogeneity between trials in the median survival ratios, higher versus lower radiotherapy dose (Fig. 2). This heterogeneity is visible in the funnel plot described above (Fig. E4), as more than the expected 5% of trials lie outside the diagonal funnel lines (4/18 trials). The degree of heterogeneity between trials of radiotherapy without chemotherapy (χ^2=45.8 on 17 degrees of freedom, p<0.001) prompted us to conduct further analyses, both of heterogeneity due to the variation in EQD2T dose differences between trials arms (Fig. E2) and by potential

confounding factors (main article). The EQD2T dose difference between trial arms ranged from 1.1 Gy to 27.2 Gy and the proportion of excess heterogeneity between studies due to dose differences in trial arms was calculated to be 28.7%, leaving over two-thirds unaccounted for. The potential confounding factors were only available at the aggregate level and thus their effects on heterogeneity could not be examined in detail (Discussion). There may also be other factors not as widely reported in the trials that could also contribute to the heterogeneity between trials.

Categorization of chemotherapy

In this study, chemotherapy was divided into two categories, concurrent and sequential. Trials defined as giving concurrent chemotherapy were those in which chemotherapy was given on the same day as any radiotherapy fraction (N=4). Of the four trials defined as giving concurrent chemotherapy, two also gave consolidation chemotherapy i.e. chemotherapy after the end of radiotherapy. Two trials were defined as giving sequential chemotherapy (N=2). In one of these trials (number 9), chemotherapy was given before radiotherapy, and there was a minimum gap of eleven days between the end of chemotherapy and the start of radiotherapy. In the other sequential trial (number 20), chemotherapy was given both before and after radiotherapy, but the number of days between chemotherapy and radiotherapy treatment was not specified. As there were so few trials giving chemotherapy (six in total), further subdivisions of chemotherapy (whether by type or timing) were not possible due to statistical power constraints.

References

1. Moher D, Liberati A, Tetzlaff J, Altman DG. Preferred reporting items for systematic reviews and meta-analyses: the PRISMA statement. Journal of clinical epidemiology. 2009;62(10):1006-12. Epub 2009/07/28.
2. Routh A, Hickman BT, Khansur T. Report of a prospective trial - Split course versus conventional radiotherapy in the treatment of non small cell lung cancer. Radiation Medicine - Medical Imaging and Radiation Oncology. 1995;13(3):115-9.
3. Siannis F, Barrett JK, Farewell VT, Tierney JF. One-stage parametric meta-analysis of time-to-event outcomes. Stat Med. 2010;29(29):3030-45. Epub 2010/10/22.
4. Brookmeyer R, Crowley J. A Confidence Interval for the Median Survival Time. Biometrics. 1982;38:29-41.
5. Sterne J, Harbord R. Funnel plots in meta-analysis. The Stata Journal. 2004;4(2):127-41.
6. Egger M, Davey Smith G, Schneider M, Minder C. Bias in meta-analysis detected by a simple, graphical test. BMJ (Clinical research ed). 1997;315(7109):629-34. Epub 1997/10/06.

Table E1. Trials included in meta-analysis, ordered according to EQD2T* radiotherapy dose difference between trial arms: patient characteristics, outcomes and any excluded trial arms

Trial no.[†]	Author, year (ref)	Randomization years	Country	No. patients	Age (years)	Percent male	Stage: System used, percent in each group	Percent SCC	Trial arm [‡]	Median survival in trial arm (months)	Median survival ratio, higher to lower dose	Excluded trial arms
1	Zhan 2007 (35)	2000 - 2005	China	159	Mean: 59	75	IIIA[§] : 42%<br>IIIB: 58%	38	A<br>B<br>C	16.4<br>15.4<br>17.6	0.9<br>0.9<br>baseline	In three included arms patients received the same chemotherapy; in a fourth excluded arm, patients received radiotherapy only
2	Saunders 1999 (19)	1990 - 1995	UK, Germany, Sweden	563	31-50: 7%<br>51-60: 24%<br>61-70: 43%<br>71+: 26%	77	IA[§] : 6%<br>IB: 24%<br>II: 7%<br>IIIA: 38%<br>IIIB: 23%<br>Unknown: 3%	82	A<br>B	16.5<br>13.0	1.3	-
3	Schild 2002 (20)	1994 - 1999	US	234	NS	62	IIIA[§] : 52%<br>IIIB: 48%	37	A<br>B	14.0<br>15.0	0.9	-
4	Bonner 1998 (21)	1992 - 1993	US	67	Median: 64	63	IIIA[§] : 60%<br>IIIB: 40%	63	A<br>B	8.2<br>11.6	0.7	In two included arms patients received radiotherapy only; in a third excluded arm, patients also received chemotherapy
5	Cox 1990 (22)	1983-1987	US	516	<60: 36%<br>60-70: 44%<br>>70: 19%	76	RTOG [¦]<br>II: 12%<br>III: 55%<br>IV: 33%	54	A<br>B<br>C<br>D<br>E	10.5<br>8.7<br>10.0<br>6.3<br>9.2	1.1<br>0.9<br>1.1<br>0.7<br>baseline	-
6	Slawson 1988 (23)	1982 - 1986	US	120	NS	NS	AJCC 1977 [¶]<br>III: 97%<br>IV: 3%	69	A<br>B	10.0<br>12.0	0.8	-
7	Baumann 2011 (17)	1997 - 2005	Germany, Poland, Czech Republic	406	Median: 66 (range 38 - 87)	90	UICC 1992<br>I: 10%<br>II: 6%<br>IIIA: 38%<br>IIIB: 46%<br>Unknown: 0%	58	A<br>B	15.6<br>15.6	1.0	-
8	Fu 1994 (24)	1990 - 1992	China	105	Median: 61 (range 21-78)	86	UICC 1989<br>I + II: 11%<br>IIIA: 55%<br>IIIB: 35%	64	A<br>B	13.1<br>7.3	1.8	-
9	Belani 2005 (25)	1998 - 2001	US	119	Median: 65 (range 40-77)	61	Stages IIIA, IIIB [§ #]	42	A<br>B	20.3<br>14.9	1.4	-
10	Sapkota 2013 (26)	NS	India, Nepal	30	NS	NS	Unresectable but not metastatic [**]	NS	A<br>B	18.0<br>15.0	1.2	-

Trial no.[†]	Author, year (ref)	Randomization years	Country	No. patients	Age (years)	Percent male	Stage: System used, percent in each group	Percent SCC	Trial arm [‡]	Median survival in trial arm (months)	Median survival ratio, higher to lower dose	Excluded trial arms
11	Sause 2000 (27)	1989 - 1992	US, Canada	301	NS	70	AJCC 1988<br>II: 6%<br>IIIA: 45%<br>IIIB: 49%	44	A<br>B	12.0<br>11.4	1.1	In two included arms patients received radiotherapy only; in third excluded arm, patients also received chemotherapy
12	Reinfuss 1999 (28)	1992 - 1996	Poland	160	<50: 14%<br>50-60: 43%<br>>60: 43%	89	UICC 1987<br>IIIA: 34%<br>IIIB: 66%	90	A<br>B	12.0<br>9.0	1.3	One arm excluded as patients received no radiotherapy
13	Zajusz 2006 (32)	2001 - 2006	Poland	53	Mean: 62	NS	Stages II-III [§] [**]	NS	A<br>B	13.7<br>14.1	1.0	-
14	Bradley 2015 (6)	2007 - 2011	US, Canada	424	Median: 64	59	AJCC [¦]<br>IIIA: 65%<br>IIIB: 35%	44	A<br>B	20.3<br>28.7	0.7	-
15	Ball 1999 (18)	1989 - 1995	Australia	99	Median: ~65 (range 40-79)	77	UICC 1987<br>I: 18%<br>II: 4%<br>IIIA: 49%<br>IIIB: 28%<br>IV: 1%	85	A<br>B	14.4<br>13.8	1.0	In two included arms patients received radiotherapy only, in two excluded arms, patients also received chemotherapy but differing regimens
16	Zhu 2000 (36)	1993 - 1996	China	70	Median: 48	87	UICC 1989<br>Inoperable I, II, IIIA, or IIIB [**]	60	A<br>B	32.0<br>16.3	2.0	-
17	Cheng W 2007 (34)	1999 - 2002	China	81	Mean: 60	74	UICC 1997<br>IIIA: 51%<br>IIIB: 49%	47	A<br>B	30.6<br>13.4	2.3	-
18	Cheng J 2004 (33)	1995 - 1998	China	74	Median: 54	78	UICC 1992<br>IIIA: 78%<br>IIIB: 22%	71	A<br>B	29.9<br>17.5	1.7	-
19	Wang 2005 (29)	2001 - 2003	China	86	Median: 48	66	UICC 1997<br>IIIA: 45%<br>IIIB: 55%	41	A<br>B	19.8<br>11.4	1.7	-
20	Yu 2014 (31)	2009 - 2011	China	60	Median: 64 (range 36-74)	78	AJCC 2007<br>IIIA: 52%<br>IIIB: 48%	65	A<br>B	20.5<br>17.8	1.2	-
21	Wang 2008 (30)	2004 - 2006	China	68	Median: ~50	68	UICC 1997<br>IIIA: 41%<br>IIIB: 59%	37	A<br>B	18.3<br>12.6	1.5	-

Abbreviations : SCC = squamous cell carcinoma; NS = not specified. RTOG = Radiation Therapy Oncology Group. AJCC = American Joint Committee on Cancer. UICC = Union for International Cancer Control.

[*] EQD2T is time-corrected equivalent dose in 2 Gy fractions.

† Trials numbered according to ascending dose difference between trial arms

[‡] Study arms presented in order of descending difference in EQD2T between trial arms

[§] No staging system provided

[¦] No year of staging system provided

[¶] AJCC 1977 staging used with an RTOG modification for Stage IV

[#] Patients eligible if stages IIIA and IIIB. Percent of patients by stage only given for T and N separately.

** Percent of patients by stage not specified

Table E2. Trials included in meta-analysis, ordered according to EQD2T* radiotherapy dose difference between trial arms: protocol treatment information, radiotherapy and chemotherapy

		Radiotherapy dose/fractionation							EQD2T*		Chemotherapy
Trial no.[†]	Author, year (ref)	Trial arm[‡]	Total dose (Gy)	Dose per fraction (Gy)	No. fractions	No. fractions per day/ days of RT per week	Split course RT: length of break	Average total treatment time (days) [§]	EQD2T* (Gy)	EQD2T difference between arms (Gy)	Protocol treatment
1	Zhan 2007 (35)	A	65.0 - 70.0	1.6	~42 ¦	2/5	A: 10 days	38.6	56.2	2.4	During and after RT: two cycles each cisplatin and etoposide.
		B	65.0 - 70.0	2.0	~56 ¦	2/5		47.2	54.9	1.1	
		C	65.0 - 70.0	1.2	~34 ¦	1/5		38.8	53.8	baseline	
2	Saunders 1999 (19)	A	54.0	1.5	36	3/7	-	12.0	51.8	2.1	-
		B	60.0	2.0	30	1/5		41.6	49.7		
3	Schild 2002 (20)	A	60.0	2.0	30	1/5		41.6	49.7	2.5	During RT: Etoposide 100mg/m², cisplatin 39mg/m²
		B	60.0	1.5	40	2/5	B: 2 weeks	41.6	47.2		
4	Bonner 1998 (21)	A	60.0	2.0	30	1/5		41.6	49.7	2.5	-
		B	60.0	1.5	40	2/5	B: 2 weeks	41.6	47.2		
5	Cox 1990 (22)	A	79.2	1.2	66	2/5	-	45.8	61.5	12.3	-
		B	74.4	1.2	62	2/5		43.0	58.4	9.2	
		C	69.6	1.2	58	2/5		40.2	55.4	6.2	
		D	64.8	1.2	54	2/5		37.4	52.3	3.1	
		E	60.0	1.2	50	2/5		34.6	49.2	baseline	
6	Slawson 1988 (23)	A	60.0	2.0	30	1/5	-	78.0	49.7	3.2	-
		B	60.0	5.0	12	1/1		41.6	46.5		
7	Baumann 2011 (17)	A	60.0	1.5	40	3/5	-	19.2	57.5	3.9	-
		B	66.0	2.0	33	1/5		45.8	53.6		
8	Fu 1994 (24)	A	69.6	1.2-1.3	56-62	2/5	-	45.8	52.3	4.0	-
		B	63.9	1.8-2.0	32-26	1/5		50.0	48.3		
9	Belani 2005 (25)	A	57.6	1.5 - 1.8 [¶]	36	3/5	-	17.8	55.7	4.1	Before RT: Carboplatin, area under the time-concentration curve 6 mg/mL/min; paclitaxel, 225 mg/m²
		B	64.0	2.0	32	1/5		45.8	51.6		
10	Sapkota 2013 (26)	A	60.0	1.5	40	2/5	-	27.6	54.2	4.5	During RT: Cisplatin weekly 30mg/m²
		B	60.0	2.0	30	1/5		41.6	49.7		
11	Sause 2000 (27)	A	69.6	1.2	58	2/5	-	40.2	55.4	5.7	-
		B	60.0	2.0	30	1/5		41.6	49.7		
12	Reinfuss 1999 (28)	A	50.0	2.0	25	1/5		34.6	43.2	6.8	-
		B	40.0	4.0	10	1/5	B: 4 weeks	41.6	36.4		
13	Zajusz 2006 (32)	A	72.0	1.8	40	1/7	-	40	61.3	7.8	-
		B	72.0	1.8	40	1/5		55.6	53.5		
14	Bradley 2015 (6)	A	74.0	2.0	37	1/5	-	51.4	58.8	9.1	Half of patients in arms A and B: Sequential and concurrent: weekly paclitaxel (45mg/m²) and carboplatin (area under the time-concentration curve 2); Other half of patients in arms A and B: Sequential and concurrent: cetuximab; weekly paclitaxel (45mg/m²) and carboplatin (area under the time-concentration curve 2)
		B	60.0	2.0	30	1/5		41.6	49.7		

		Radiotherapy dose/fractionation							EQD2T*		Chemotherapy
Trial no.[†]	Author, year (ref)	Trial arm[‡]	Total dose (Gy)	Dose per fraction (Gy)	No. fractions	No. fractions per day/ days of RT per week	Split course RT: length of break	Average total treatment time (days) [§]	EQD2T* (Gy)	EQD2T difference between arms (Gy)	Protocol treatment
15	Ball<br>1999 (18)	A<br>B	60.0<br>60.0	2.0<br>2.0	30<br>30	2/5<br>1/5	-	20.6<br>41.6	60.0<br>49.7	10.3	-
16	Zhu<br>2000 (36)	A<br>B	76.0<br>70.0	1.8 - 2.0 [#]<br>2.0	40<br>35	first 20: 1/5 [#]<br>1/5	-	55.6<br>48.6	68.8<br>56.2	12.6	-
17	Cheng W<br>2007 (34)	A<br>B	70.0<br>68.0 - 72.0	1.5 - 2.0 [**]<br>2.0	40<br>34 - 36	first 20: 1/5 [**]<br>1/5	-	41.6<br>48.6	68.8<br>56.2	12.6	-
18	Cheng J<br>2004 (33)	A<br>B	64.0<br>65.0 - 70.0	1.2 - 2.0 [††]<br>2.0	40<br>33 - 35	first 20: 1/5††<br>1/5	-	41.6<br>45.8	68.8<br>55.6	13.2	-
19	Wang<br>2005 (29)	A<br>B	64.0 - 70.0<br>66.0 - 70.0	2.0 - 5.0 [‡‡]<br>2.0	26<br>33-35	first 20: 1/5 [‡‡]<br>1/5	-	40.4<br>45.8	68.8<br>53.6	15.2	-
20	Yu<br>2014 (31)	A<br>B	65.0<br>60.0	2.5 - 3.0<br>2.0	25<br>30	1/5<br>1/5	-	34.6<br>34.6	68.8<br>53.2	15.6	Before and after RT: vinorelbine 25mg/m^2 and cisplatin 40mg/m^2
21	Wang<br>2008 (30)	A<br>B	80.0<br>66.0 - 70.0	2.0 - 5.0 [§§]<br>2.0	34<br>33-35	first 30: 1/5 [§§]<br>1/5	-	50.4<br>45.8	80.8<br>53.6	27.2	-

Abbreviations : RT = radiotherapy.

[*] EQD2T = time-corrected equivalent dose in 2 Gy fractions.

[†] Trials numbered according to ascending dose difference between trial arms

[‡] Trial arms presented in order of descending difference in EQD2T between trial arms

[§] Calculated as an average of the total days of treatment, assuming equal numbers of patients started regimen on each of five days (Monday to Friday)

[¦] Number of fractions not indicated, so number of fractions estimated by dividing total dose by total treatment time

[¶] The first and third fractions of each day were 1.5 Gy, the second fraction was 1.8 Gy

[#] First 20 fractions conventional irradiation at 2 Gy per fraction, final 20 fractions delivered twice daily at 1.8 Gy per fraction

[**] First 20 fractions conventional irradiation at 2 Gy per fraction, final 20 fractions delivered twice daily at 1.5 Gy per fraction

[††] First 20 fractions conventional irradiation at 2 Gy per fraction, final 20 fractions delivered twice daily at 1.2 Gy per fraction

[‡‡] First 20 fractions conventional irradiation at 2 Gy per fraction, final six fractions at 4-5 Gy per fraction, final six fractions given every other day

[§§] First 30 fractions conventional irradiation at 2 Gy per fraction, final four stereotactic fractions at 5Gy per fraction, final four fractions given every other day

Figure E3. EQD2T* radiotherapy dose in individual arms of trials comparing different radiotherapy regimens, with or without chemotherapy, by year trial started†

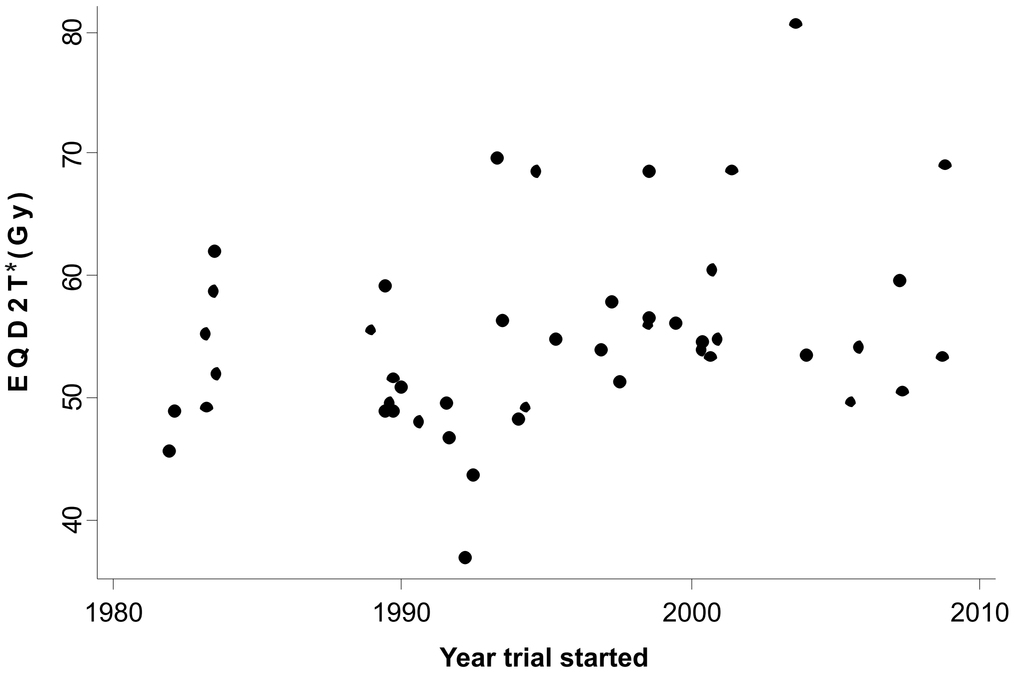

* EQD2T is time-corrected equivalent dose in 2 Gy fractions

†Average increase in EQD2T per year: 0.31Gy (95% CI 0.02 - 0.61 Gy, p=0.04)

Table E3. Correlation of geographical region, average age at trial entry, year trial started, and percent of patients with squamous cell carcinoma *

	Average age at trial entry	Geographical region	Year trial started	Percent with squamous cell cancer
Average age at trial entry	1.00			
Geographical region	0.69	1.00		
Year trial started	-0.71	-0.70	1.00	
Percent with squamous cell cancer	0.23	0.46	-0.45	1.00

* Trial number 13 is excluded from this analysis as it is missing information on histological subtype

Table E4. Variation in pooled median survival ratio, higher versus lower EQD2T dose, by average age at trial entry, geographical region, year trial started, and percent of patients with squamous cell carcinoma *

Exploratory factor	Group	Ratio † (95% CI)	p for heterogeneity
**Average age at trial entry**	≤60	1	0.4
	60+	0.87 (0.65 - 1.17)	
	Average age in trial not known	0.76 (0.50 - 1.16)	
**Geographical region**	China	1	0.008
	Elsewhere	0.66 (0.49 - 0.90)	
**Year trial started**	1980s	1	0.2
	1990s	1.13 (0.92 - 1.39)	
	2000s	0.78 (0.42 - 1.45)	
**Percent of patients with squamous cell carcinoma**	<50%	1	0.2
	≥50%	0.81 (0.59 - 1.12)	

* Trial number 13 is excluded from this analysis as it is missing information on histological subtype.

† Ratio of pooled estimate of median survival ratio within each exploratory factor. If there is no effect, the ratio is equal to 1. Pooled median survival ratios were estimated by variance-weighted least squares. The results for each factor are shown after adjustment for the other three.

Figure E4. Funnel plot of median survival ratios and standard errors of randomized treatment comparisons in which radiotherapy is given without chemotherapy

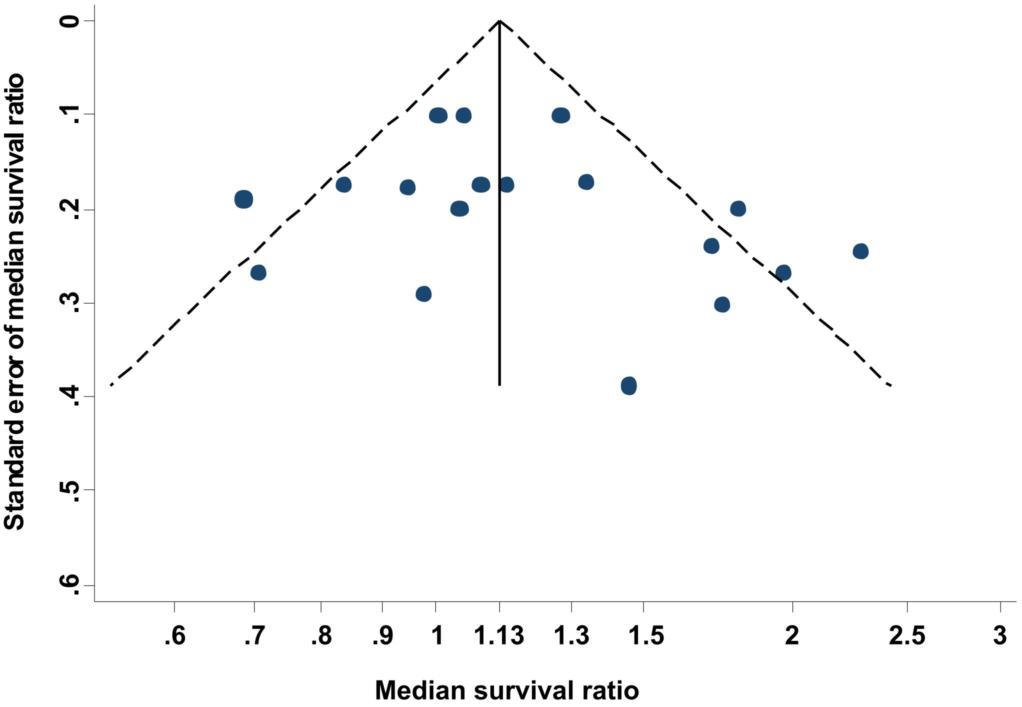

Each point on this plot represents the standard error of the median survival ratio for a randomized dose comparison, plotted against the median survival ratio. The vertical solid line represents the pooled estimate of all randomized comparisons. The diagonal, broken lines represent the area around the line within which 95% of studies with the standard error indicated on the y-axis are expected to fall, given random variation only. The bigger the trial the closer to the top of the funnel it lies and the smaller the expected difference between its median survival ratio and the pooled effect. Small study effects are assessed by the degree of asymmetry in the points to the right or left of the line toward the bottom of the plot. If there were a high degree of asymmetry, there would be many points located in the bottom right or bottom left of the plot. In this study that is not the case. However, there are four studies (22%) that lie outside of the funnel (one to the left, three to the right), while only 5% (1 study) is expected to lie outside the plot if the only variation present was random. This shows that there is heterogeneity between the studies.
